# The Finite Absorption Time Concept Guiding Model Informed Drug & Generics Development in Clinical Pharmacology

**DOI:** 10.1007/s11095-025-03878-4

**Published:** 2025-06-05

**Authors:** Panos Macheras, Athanasios A. Tsekouras, Sergio Sánchez-Herrero, Kosmas Kosmidis

**Affiliations:** 1 Faculty of Pharmacy, National and Kapodistrian University of Athens, Athens, Greece; 2 PharmaInformatics Unit, ATHENA Research Center, Athens, Greece; 3 Department of Chemistry, National and Kapodistrian University of Athens, Athens, Greece; 4 Department of Computer Science, Multimedia and Telecommunication, Universitat Oberta de Catalunya, Barcelona, Spain; 5 Simulation Department, Empresarios Agrupados Internacional S.A, Madrid, Spain; 6 Division of Theoretical Physics, Physics Department, Aristotle University of Thessaloniki, 54124 Thessaloniki, Greece

**Keywords:** Bioequivalence, Finite absorption time, IVIVC, Oral drugs, Pharmacokinetics, Physiologically based finite time pharmacokinetic models

## Abstract

**Purpose:**

To show the implications of the incorporation of the Finite Absorption Time (F.A.T.) concept in drug development plans and in generics development and assessment and to examine regulatory implications.

**Methods:**

Reexamining and reanalyzing published pharmacokinetic data using the pertinent models that are based on F.A.T.

**Results:**

Comparing absorption metrics, old and new ones, shows distinct advantages and better accuracy for those based on the F.A.T. concept.

**Conclusion:**

The proposed approaches can be applied successfully in all phases of drug/generics development and guide changes in their strategy and in the relevant regulatory framework.

## Introduction

Clinical pharmacology is inherently a translational discipline engaged in the experimental and observational study of the disposition and effects of drugs in humans. Until the 1960 s variability in drug response was always associated with the patient in accord with Sir William Osler’s variability principle which says “Variability is the law of life, and as no two faces are the same, so no two bodies are alike, and no individuals react alike and behave alike under the abnormal conditions which we know as disease” [1, 2]. In the early 1970 s, however, it was realized that a variable or poor response to a therapeutic agent may not have its origin in the patient; it may be due to a formulation defect in the drug product administered [3, 4]. The introduction of the bioavailability concept by the U.S. Food and Drug Administration [5] in 1977, was a logical consequence for the protection of public health. Since then, the intertwined emergence of bioavailability and clinical pharmacology lead to the incorporation of advanced quantitative methods into regulatory procedures, the so-called Model Informed Drug Development (MIDD) initiative, which modernize the development and regulation of drugs, as well as their use in the practice of medicine [6–8]. Overall, MIDD approaches rely on pharmacokinetic, pharmacodynamic and pharmacometrics studies in all phases of drug development [6, 8] as well as the individualization of drug therapy [7].

In the field of oral drug absorption, Dost described in his first two pharmacokinetics books published in 1953 and 1968 [9, 10] the blood drug concentration as a function of time assuming first-order absorption kinetics [11], which implies that drug absorption runs for infinite time. However, the recent analysis of oral drug absorption based on the finite absorption time (F.A.T.) concept [12] and the relevant physiologically based finite time pharmacokinetic (PBFTPK) models developed, provided meaningful and reliable estimates for the duration of drug absorption, *τ*, as well as for the corresponding drug input rate(s) [12–15]. We also uncovered the real meaning of the fundamental parameters *C*_max_ and ${\left[AUC\right]}_{0}^{\infty }$ in the light of the physiologically sound F.A.T. concept [14]. This realization coupled with the analytical power of PBFTPK models providing new parameters for oral drug absorption, leads to the emergence of a new world in all phases of drug and generics development. In this context, we propose i) to alter the strategy in the early phases and Phase I studies of drug development enriching the Phase I studies with an absolute bioavailability study, ii) to utilize PBFTPK models in the analysis of pharmacokinetic, pharmacodynamic, pharmacometric data in Phase II and III studies, iii) to change the strategy of generics development and iv) to apply model-dependent and model independent approaches for bioequivalence assessment based on the F.A.T. concept.

## Drug Development

### Early Phases and Phase I Studies

**Fig. 1:**
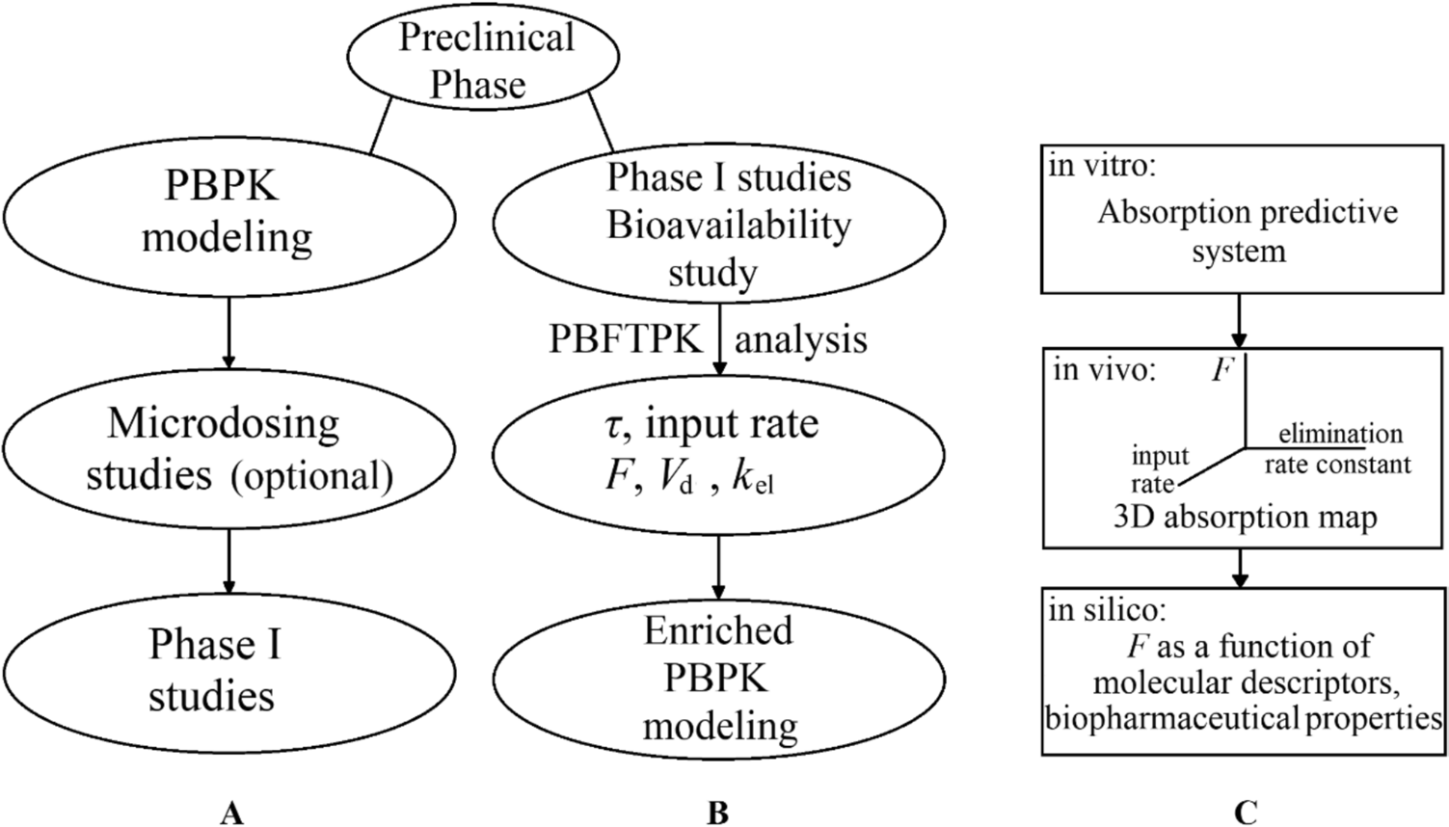
(**A**) Currently used studies in early phase of drug development in chronological order. (**B**) Proposed additional F.A.T. driven Phase I studies followed by enriched PBPK modeling work. (**C**) Future developments: Towards a predictive experimental device, the *“3D absorption map”* era and the predictive absorption models based on molecular structure using machine learning techniques.

**Fig. 2:**
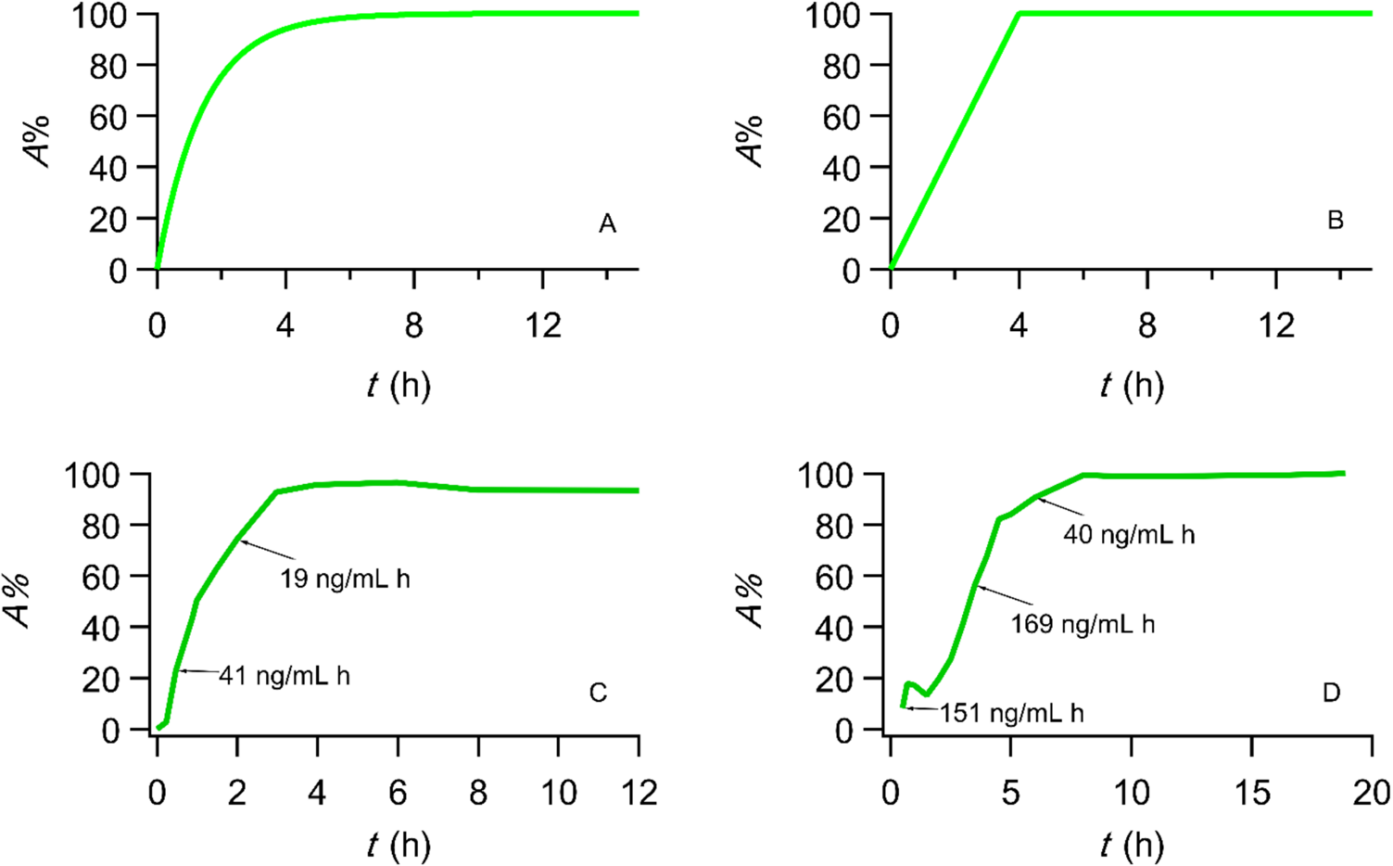
A paradigm shift in oral drug absorption. (**A**) Currently, the percent absorbed A% *versus* time curves follow a mono-exponential pattern based on the prevailing hypothesis of first-order absorption kinetics. (**B**) Linear percent absorbed *versus* time plot in accord with the F.A.T. concept assuming one input stage [27]. (**C**) Percent absorbed *versus* time curve for almotriptan with two linear absorption segments (redrawn from Fig. 8 in [15] using Eq. 6 from Ref. [27]). (**D**) Percent absorbed *versus* time curve for cyclosporine reference product under fed conditions exhibiting three linear absorption segments (data replotted from Fig. 9D in [15] using Eq. 6 from Ref. [27]).

**Fig. 3:**
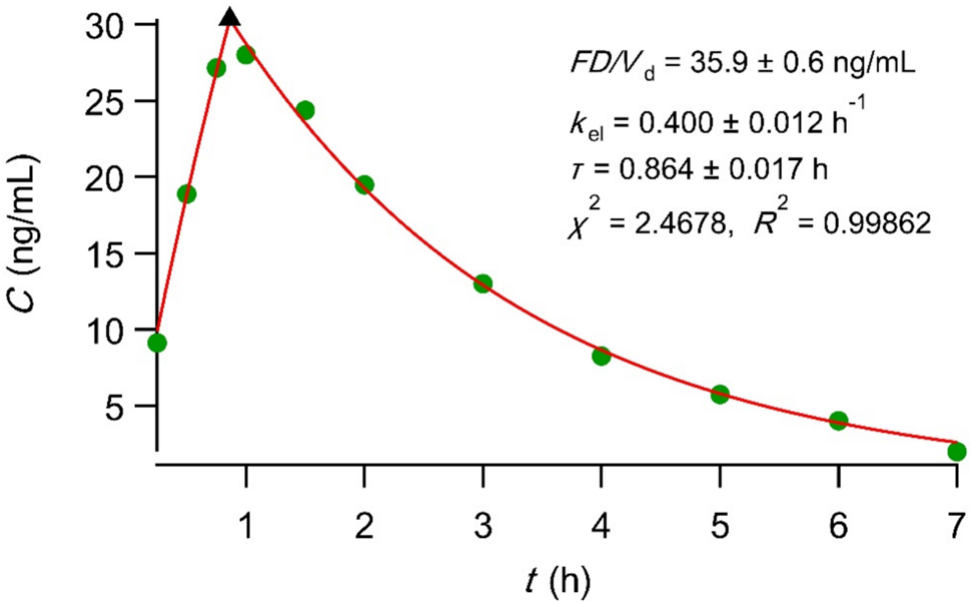
Best fit results of the PBFTPK model [15] with one input stage assuming one-compartment model disposition to alendronate experimental blood data [29]. The symbol ▲ denotes the end of the absorption processes. *V*_*d*_ is the volume of drug distribution and *k*_*el*_ is the elimination rate constant.

Upon completion of the preclinical phase, *in silico* approaches [16], including physiologically based pharmacokinetic **(**PBPK) modeling, are frequently applied, which are followed by Phase I studies to find the optimal dose and assess safety (Fig. 1A). The PBPK models are used for predicting drug’s absorption, distribution, metabolism and excretion based on the physiological, physical, and chemical descriptions of the phenomena involved. For PBPK models focusing on gastrointestinal absorption, the preclinical data of the biopharmaceutical properties solubility, permeability and factors such as dose and drug particle size associated with drug dissolution are of extreme importance. In some cases, big pharmaceutical companies developing poorly soluble drugs, e.g., anticancer, like to know the extent of drug’s absorption prior to Phase I studies and the huge investment and perform laborious microdosing or phase zero studies [17–23]. Thus, the current scenario of early phases and Phase I studies is depicted in Fig. 1A in chronological order. In the PBPK modelling studies first-order kinetics is being used since the absorption rate constant *k*_a_ is linked with the effective permeability *P*_eff_ and radius *R* of the gastrointestinal lumen, namely, *k*_a_ = 2 *P*_eff_/*R*, while finite transit times are allocated in the drug movement among the compartments of the gastrointestinal tract [16]. Thus, the predicted % absorbed *versus* time curves of these studies have a mono-exponential pattern (Fig. 2A). This plot has its roots in the Wagner-Nelson and Loo-Riegelman papers of the 1960 s [24–26], which have been transformed and used today in deconvolution software. However, the equations describing the percent of drug absorbed as a function of time quoted in refs [24–26] were modified in Ref [27] in accord with the F.A.T. concept; we have also proved that this plot is either linear (Fig. 3B) or multilinear (Fig. 3C, D), if more than one linear drug input rates are encountered.

The advances in the understanding of gastrointestinal absorption based on the F.A.T. concept and the use of PBFTPK models so far point to the enriched scenario for Phase I studies depicted in Fig. 1B. According to this scheme, the classical Phase I studies to find the optimal dose and assess safety are supplemented with an oral absolute bioavailability study applying for example blood sampling and urine collection. From the analysis of blood concentration, time data, the characteristics of drug absorption are estimated using the PBFTPK models, namely, i) the number of absorption stages, ii) the corresponding drug input rates and iii) the duration of each stage *τ*_*i*,_ as well as the total duration of absorption, *τ* (Fig. 2B). These data will be useful for simulating dosage regimen designs for therapeutic purposes. Additionally, useful conclusions can be drawn for the formulation development plan. For example, the analysis of a typical BCS Class II drug carbamazepine [28] reveals a long duration of absorption (*τ* > 16 h), which rules out the development of a sustained release formulation, a desirable trait due to its chronic use. The analysis of the absolute bioavailability study can be performed using Eqs. 1 and 2 (see the derivation of Eq. 1 in the appendix).

1 $${\left[AUC\right]}_{\tau }^{\infty }=\frac{FD-{Q}_{el}(\tau )}{CL}$$

Dividing Eq. 1 with the fundamental equation ${\left[AUC\right]}_{0}^{\infty }=FD/CL$, one can derive the following expression for the bioavailable fraction, *F*:

2 $$F=\frac{{Q}_{\text{el}}(\tau )}{D}{\left(1-\frac{{\left[AUC\right]}_{\tau }^{\infty }}{{\left[AUC\right]}_{0}^{\infty }}\right)}^{-1}$$

where *D* is the drug dose, *Q*_el_(*τ*) is the amount eliminated up to time *τ*, and *CL* is the drug clearance. We present a relevant example using the alendronate blood and urine data reported in the literature [29]. Figure 3 shows the fitting of the PBFTPK model [15] with one input stage assuming one compartment model disposition to drug blood data. An estimate for *Q*_el_(*τ*) equal to 175 μg was derived from the urine data reported in Fig. 2B of [29] using interpolation at time *τ* = 0.86 h. The estimate for *F* using Eq. 2 was found equal to 175 μg/70 mg [1-(83–13.8)/83]^−1^ = 1.5%, while the plateau value of the urine excretion 727 μg (Fig. 2B in [29]) divided by the dose 70 mg gives an estimate 1.03%; these estimates for *F* are very close to the literature value, 0.7% [30]. In this example, the estimation of *Q*_el_(*τ*) was rather simple since alendronate is not metabolized and is eliminated via renal excretion [30]. For drugs metabolized, however, measurements for the eliminated metabolite(s) are required for the correct estimation of *Q*_el_(*τ*)*.*

Another useful approach for the estimation of absolute bioavailability relies on the so called “Semi simultaneous approach” [31–33], which can be easily adapted in the realm of F.A.T. concept by administering the intravenous bolus dose beyond the end of the absorption process, *τ*. We re-analyzed the LiCl rat data in [32] by fitting the PBFTPK model with one input stage and two compartment disposition to the entire set of intraperitoneal-intravenous data, adjusting the duration of absorption for the intraperitonial administration in accord with the sampling design, while the duration of the intravenous administration was set equal to 36 s. The estimated absolute bioavailability of the intraperitonially administered formulation based on “the concentration maxima” of the two administrations was found equal to one very close to the estimate derived from the conventional methodology based on the comparison of areas under the curve [32].

Overall, the estimates for *τ*, number-duration-magnitude of drug input rates, *F* and *V*_d_ values derived from the absolute bioavailability study will formulate the basics for drug development. These results show that the microdosing studies quoted in Fig. 1A can be replaced by the bioavailability study as part of the Phase I studies (Fig. 1B). Although investigators during Phase I studies are looking at the effects of the medication on about 20 to 80 healthy volunteers to figure out the highest dose humans can take without serious side effects, the performance of the absolute bioavailability study will allow this consideration to be based on the actual bioavailable dose. Hence, Phase I studies for oral drugs can be renamed “*Phase I: First dose in man studies/Absolute bioavailability assessment*” since the estimate for *F* will be in the heart of all phases of drug development*.* In other words, the strategy of drug development will change as shown in Fig. 1B. According to the scenario depicted in Fig. 1B, the estimates derived from this analysis will be used in an enriched PBPK modeling exercise focusing on the optimization of formulation in terms of the dose and particle size of drug. Thus, the estimate for *τ* can be applied to finite time dissolution functions, e.g., Noyes-Whitney and the Weibull equations [34] used in the enriched PBPK work (Fig. 2B), for studying the effect of drug’s particle size and dose variation on the predicted blood concentration, time profile. Besides, the estimate for *τ* will guide the enriched PBPK modeling work; for example, if *τ* is less than 5 h, drug absorption takes place in the small intestine and colon absorption is ruled out. It should also be mentioned that the estimate for the volume of drug’s distribution (derived from the *FD/V*_d_ estimate of the PBFTPK model fitting and the estimate for *F* from the absolute bioavailability study) allows its use in the PBPK modeling work and the expression of drug blood levels in real, clinically relevant units. It can be anticipated that the use of PBFTPK models for the analysis of pharmacokinetic data coupled with the methods for the estimation of *F* will be applied to many drugs. Plausibly, this will progressively lead to the creation of a *“3D absorption map”* with the *xy* plane corresponding to the input rate and the elimination rate constant, which drives the elimination rate, while the *z*-axis will represent the *F* estimates (Fig. 1C). Plausibly, the *“3D absorption map”* will be used as an advanced biopharmaceutic-pharmacokinetic system, e.g., biowaivers are in the region *F* > 0.90. Consecutively, this advancement will ultimately lead to *in silico* predictive absorption models since the input rate corresponds to the estimate of the ratio (*FD*/*V*_d_)/*τ* while the elimination rate constant, *k*_el_ controls the elimination of drug. All parameters are strongly associated with drugs’ molecular descriptors, physicochemical and biopharmaceutical properties. In other words, the strategy of development in the early phases for the estimation of absolute bioavailability will move progressively to “*in silico*” approaches utilizing machine learning techniques too (Fig. 1C).

The emergence of absolute bioavailability estimation with minimal intravenous sampling mentioned above will result in the reduction of intravenous administrations during drug development. In parallel, the development of oral drugs will be propelled and their clinical use will be substantiated from pharmacokinetic, pharmacodynamic studies centered around the estimated bioavailable dose. Thus, these advances can find favorable ground in anticancer chemotherapy, whereas the intravenous-to-oral switch has been under discussion for several years [35]. This is also in line with the current tendency for deintensification in cancer care [36] since oral formulations have lower bioavailability than the intravenous solutions. However, the lower bioavailability of the oral formulations may result in more frequent oral administration. Under these circumstances, the use of anticancer-milk oral formulations with enhanced bioavailability properties can be considered [37]. It is advisable therefore, FDA to grant GRAS (Generally Regarded As Safe) status for the use of milk in anticancer and paediatric formulations considering the risk/benefit ratio and the fact that milk is or can be a daily ritual for the oncology patient. According to a recent study [38], WHO has granted GRAS status to milk. Anticancer chemotherapy based on oral drug administration will certainly improve the life of oncology patients avoiding the hospitalization for the intravenous drug administration. Such a development will underscore the rise of “greener oncology therapy”.

### Phases II and III

**Fig. 4:**
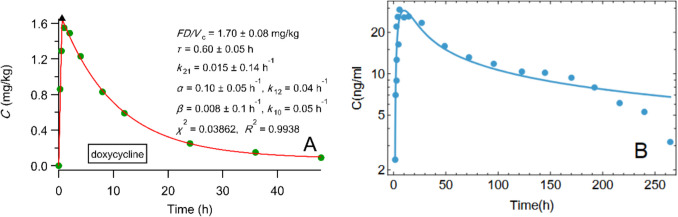
(**A**) PBFTPK model nonlinear fitting [13] with one input stage and two-compartment model disposition to doxycycline data [45]. The symbol ▲ denotes the end of the absorption process. (**B**) Fitting using a custom code written in Wolfram Language (Mathematica version 14.2) to methylprednisolone data [46] after the intramuscular administration of methylprednisolone acetate, which is a pro-drug of methylprednisolone; the equation $C\left(t\right)={e}^{{-k}_{\text{el}}t}{t}^{-\lambda }\left({n}_{0}{t}^{\lambda }-kt{E}_{1}\left(\lambda , -{k}_{\text{el}}t\right)\right)$ where *E*_1_(*λ*,*x*) is the Exponential Integral function, which is the analytical solution of the ODE: $\frac{dC}{dt}=k{t}^{-\lambda }-{\text{k}}_{\text{el}}C$ was used. Best fitting results, *k* = 14.36 ng mL^−1^ h^−0.56^, *λ* = 0.44, *k*_el_ = 0.179 h^−1^ and correlation coefficient, *R*.^2^ = 0.958.

The vast majority of published pharmacokinetic, pharmacodynamic and pharmacometrics studies of Phases II and III dealing with oral drug absorption rely on first-order absorption models. In all these studies the fallacious first-order absorption rate constant governs and quantifies the rate of drug absorption. The first-order kinetic concept is being used arbitrarily in many drugs kinetic processes in the body; we quote here several examples. A first-order rate constant equal to 21 h^−1^ is being used to model bile secretion [39]; this magnitude implies a process which is 90% completed in only 6.5 min indicating that a zero-order consideration would be more physiologically relevant. Besides, when complex absorption profiles are encountered and first-order absorption kinetic models fail, stochastic approaches based on the inverse Gaussian function are employed [40]. We have shown that the PBFTPK models capture the dynamics of the complex absorption phenomena and describe the drug input rates and the duration of the input stages based on meaningful parameters [13]. Moreover, the frequently used gastro-intestinal transit time model [41] utilizes five first-order rate constants (two in series for the drug transit and three in parallel for the drug uptake) and proved superior to the classical first-order model with a lag time. Although the fitting results can be superior, the physical meaning of the five absorption rate constants is questionable due to the finite character of both drug transit and absorption processes. Also, in the field of interspecies scaling and paediatric scaling, the inverse time units of the absorption rate constant do not allow a scaling exercise. In contrast, the physiologically based meaningful input parameters of the PBFTPK models if coupled with nonlinear mixed effect modeling will allow the scaling between adult and children or species oral data. Finally*,* we briefly touch the so-called flip flop kinetics, which is used in pharmacokinetics and pharmacometrics [42, 43] under the assumption of lower value for the absorption rate constant compared to the elimination rate constant. Since this assumption is not valid under the prism of the F.A.T. concept, we present an example [44] of a successful PBFTPK model fitting (Fig. 4A) to doxycycline data [45]. We also interpreted [44] the concentration time profile of intramuscularly administered methylprednisolone acetate, which is a non-hydrosoluble pro-drug of methylprednisolone [46], Fig. 4B, using fractal kinetics [47–49] assuming a time dependent coefficient ($k{t}^{-\lambda })$ driving the input rate using the ODE, $\frac{dC}{dt}=k{t}^{-\lambda }-{k}_{\text{el}}C$. Both sets of data in [45, 46] have been described with flip flop kinetics; our results invalidate this working hypothesis.

Population analyses are an integral part of Phases II and III studies. Consequently, another important area of the PBFTPK models’ application is their use as structural models in population analysis studies. As mentioned above, the physiologically sound meaning of the parameters of the PBFTPK models will allow their scaling in interspecies and peadiatric pharmacokinetic population studies, which is not possible today because of the first-order nature of the absorption rate constant. We present preliminary results of a population PBFTPK model of abacavir in infants, toddlers and children with PhysPK® based on Zhao *et al.* data [50]. Abacavir concentration data from clinical studies in human immunodeficiency virus-infected children (*n* = 69) were used for model building based on the two-compartment PBFTPK model. Preliminary results of this first application of PBFTPK models to population analysis were obtained. From abacavir studies, data was obtained from once daily dosage regimen 16 mg kg^−1^. A population pharmacokinetic analysis was performed using a one input stage PBFTPK two-compartment model [15] with nonlinear mixed effects modelling with PhysPK®. For this preliminary model no covariance was taken into account. The preliminary results of population pharmacokinetic parameters of abacavir are shown in Table 1.

**Table 1:** Population Pharmacokinetic Parameters of Abacavir

Parameter	Estimate	Relative standard error (%)
*FD*/*V*_d_ (mg/L)	333	43.6
*τ* (h)	1.74	3
Systemic clearance, *CL* (L h^−1^)	22.1	10
Central volume of distribution, *V*_c_ (L)	24.68	3.29
Peripheral volume of distribution, *V*_p_ (L)	25.67	Fixed
Intercompartment clearance, *Q* (L h^–1^)	2.8	Fixed

**Fig. 5:**
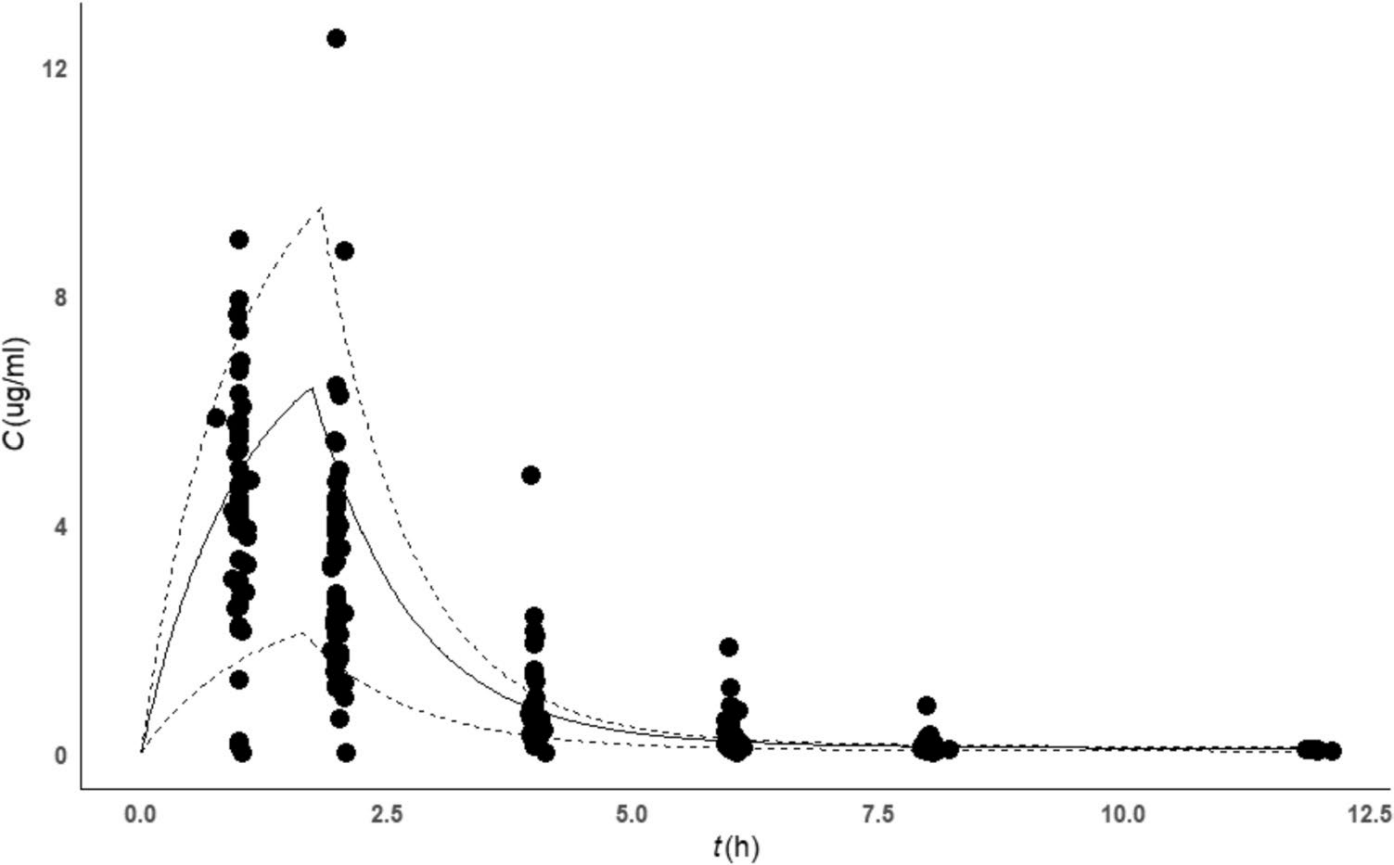
Visual predictive check in children’s following once daily dosing regimen; observed data are plotted using a circle. The dashed lines represent the 5th and 95th percentiles of simulated data (*n* = 1000). The continuous lines represent the 50^th^ percentile of simulated data (*n* = 1000).

**Fig. 6:**
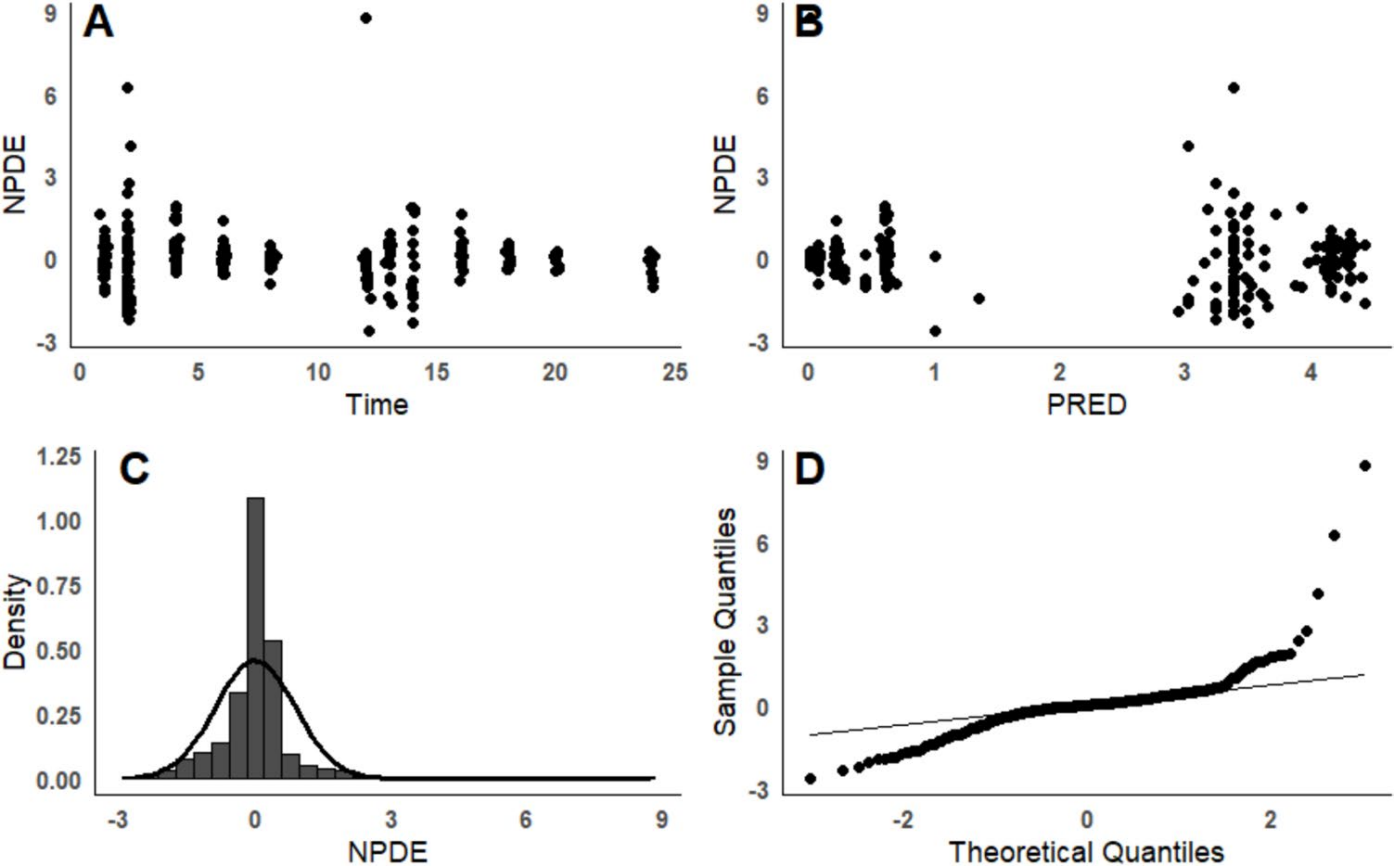
Normalized prediction distribution errors (NPDE) analysis. (**A**) NPDE *vs.* time. (**B**) NPDE *vs.* population prediction concentrations (PRED). (**C**) Histogram of the distribution of the NPDE, with the density of the standard Gaussian distribution overlaid. (**D**) QQ-plot of the distribution of the NPDE *vs.* the theoretical normal distribution.

The visual predictive check (VPC) in Fig. 5 of the final model, including all patients, showed that the observed concentrations did not align well with the predicted values, suggesting a need for model improvement. The inadequate fit might be attributed to fixing certain parameters, such as *Q* and *V*_p_, instead of estimating them, and to the need for exploring alternative PBFTPK models. Additionally, subpopulation-specific VPCs (infants, toddlers, and children) and dosing regimens (once *vs.* twice daily) may provide further insights into model refinement. The NPDE distribution and histogram in Fig. 6 indicated deviations from the expected normal distribution of prediction errors, and trends observed in the NPDE diagnostic plots against time or predicted concentrations suggest that the model needs further adjustment.

## Implications for the Development and Assessment of Generics

### Generics Development

The development of generics mostly relies on drug dissolution studies and the relevant *in vitro* *in vivo* correlations (IVIVC) [51, 52]. Almost twenty years ago we published [53] a highly cited review on drug dissolution focusing “on the realization of a relationship between dissolution and bioavailability, which initiated the drug related interest in dissolution, and progressing to the present applications of dissolution studies, with both their scientific and regulatory aspects”. The concluding remark of this work [53] was “The experience gained so far indicates that the design of a unique dissolution test to be used reliably as a prognostic tool of oral drug absorption will not appear in the near future.” Unfortunately, this conclusion is verified today despite the tremendous number of studies towards a formulation predictive dissolution testing to advance oral drug product development in the ensuing years [54–56]. Although the driving force behind these studies is the need to develop a predictive dissolution method that can be applied by pharmaceutical drug companies to facilitate marketing access for generic and novel drug products, the number of successful level A *in vitro* *in vivo* correlations (IVIVC) [57] is very limited.

We argue below that one of the most important reasons for the failed IVIVC is associated with the “taken for granted” unphysical assumption of first order drug absorption which is present in all relevant portions of the guideline [57] describing the calculation of the fraction of drug absorbed as a function of time. e.g., i) “A Level A correlation is usually estimated by a two-stage procedure: deconvolution followed by comparison of the fraction of drug absorbed to the fraction of drug dissolved”; ii) “In a linear correlation, the *in vitro* dissolution and *in vivo* input curves may be directly superimposable or may be made to be superimposable by the use of a scaling factor”; iii) “One alternative is based on a convolution procedure that models the relationship between *in vitro* dissolution and plasma concentration in a single step”; iv) “estimate the *in vivo* absorption or dissolution time course using an appropriate deconvolution technique for each formulation and subject (e.g., Wagner-Nelson, numerical deconvolution)”. The convolution, deconvolution terms in the guideline [57] are in most cases computerized synonyms of the Wagner-Nelson [24, 25] and Loo-Riegelman [26] techniques. Besides, the superimposable term above for the *in vitro* drug dissolution and *in vivo* input curve also implies first-order kinetics since dissolution is described by exponential functions based on first-order kinetic assumptions. However, the collapse of the first-order drug absorption hypothesis [12–15], the modification of Wagner-Nelson [24, 25] and Loo-Riegelman [26] equations in [27] resulted in a paradigm shift in oral drug absorption, namely, the percent absorbed *versus* time curves are either bilinear or multilinear, Fig. 2. These advances were further coupled with the physiologically sound finite dissolution time (F.D.T.) concept and resulted in the revision of IVIVC [33].

All the above call for a change in the strategy of generics development based on the following steps. The first step consists of analysis of the reference *in vivo* data using the PBFTPK models. This will provide the detailed characteristics of the drug’s absorption, i.e., number, duration, input rate for each stage of drug absorption as well as the percent absorbed *versus* time curve of the reference formulation. Useful information will be extracted from this analysis in terms of the F.D.T. considering the F.A.T. estimate derived from the fitting of PBFTPK models to the experimental data of the reference formulation as described in [28]. This will be followed by *in vitro* dissolution studies of the reference formulation in a flow through system, e.g., USP apparatus 4 using official dissolution media. This system ensures sink conditions which are in line with the sink conditions prevailing in the absorption of drugs from the gastrointestinal tract [12, 13, 15, 58]. Ideally, the drug measurements will result in a linear percent *in vitro* dissolved *versus* time curve. The next step relies on the analysis of % absorbed *versus* % dissolved plot and the quest for IVIVC and/or the scaling factor and/or the ratio of the slopes of the *in vitro* and *in vivo* lines for the reference formulation. Finally, the pharmaceutical scientist runs dissolution experiments for the generic formulation(s) under development using the experimental conditions adhering to the best IVIVC established in the previous step using the reference formulation. In summary, the steps of the new strategy for the development of generics are as follows.

1. Analysis of the *in vivo* reference formulation data using the PBFTPK models [15]
2. Construction of the percent absorbed *versus* time plot for the reference formulation [27]
3. *In vitro* dissolution studies of the reference formulation in a flow through system ensuring sink conditions
4. Correlation based on % absorbed and % dissolved data of the reference formulation considering the F.A.T. and F.D.T. time constraints [28]
5. Dissolution experiments for the generic formulation based on the previous step best results

In the spirit of the above plan, we analyze two studies [59, 60] dealing with IVIVC to demonstrate the utility of the PBFTPK modelling (step 1) for the elucidation of mesalazine and efodipine absorption processes as well as the proper construction of the percent absorbed *versus* time plot (step 2).

**Fig. 7:**
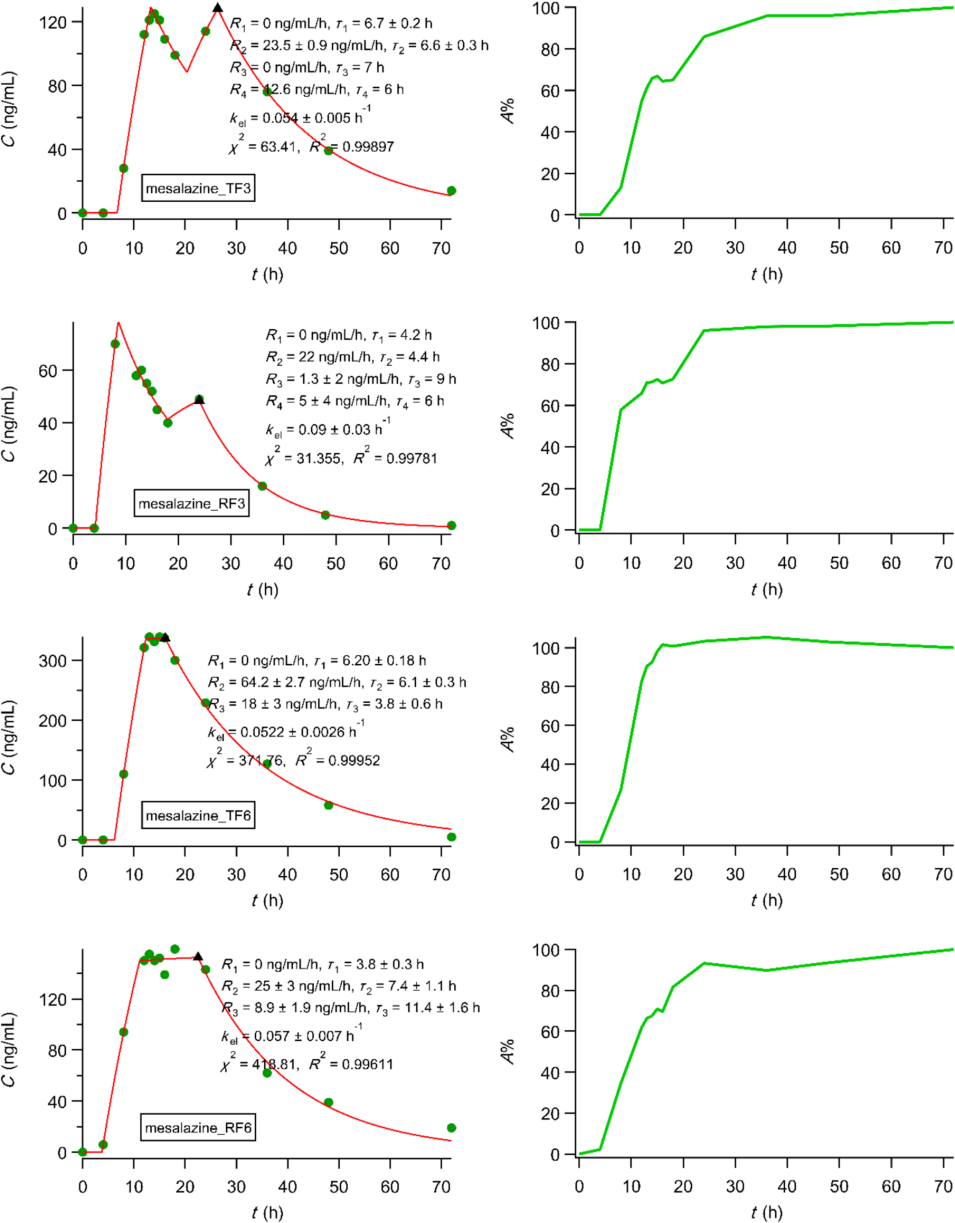

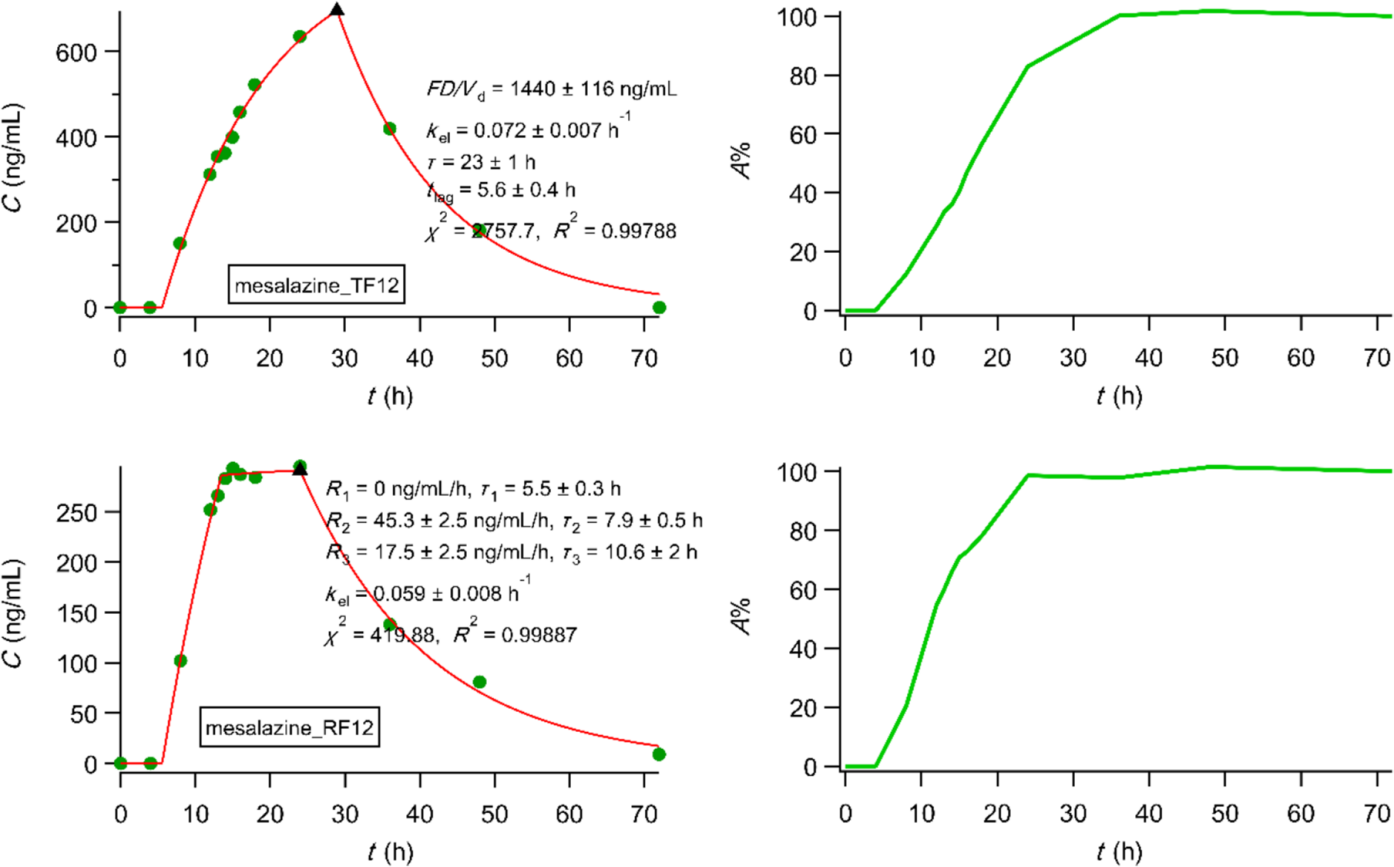
Best fitting results of PBFTPK models to mesalazine data [59]; each plot is followed by the corresponding percent absorbed *versus* time curve calculated using Eq. 2 from Ref. [27]. The meaning of symbols can be found in [59]. *R*_1_, *R*_2_, *R*_3_, *R*_4_ denote rate of input at each input stage.

We analyzed upon digitisation, using PBFTPK models [15], the bioequivalence data of mesalazine [59] colon targeting tablets of a generic development product (test formulation, TF; mesalazine 400 mg tablet) and the original product (reference formulation, RF; Asacol® 400 mg tablet) [59]. The best fitting results using the PBFTPK models [15] with one-compartment disposition for all formulations examined presented in Fig. 7 show the complexity of mesalazine absorption for the three administrations, i.e., 3, 6 and 12 tablets along with the duration of each absorption stage and the corresponding input rates. In Fig. 7 we also present the percent absorbed *versus* time plots of multilinear character indicating the end of the absorption process, which ranges from 13.6 to 38.6 h verifying the colon targeting design of the tablets. The fitting results based on the analysis of the same data using first-order absorption models were inferior, while the profiles of the percent absorbed *versus* time plots were exponential. Overall, the dissolution data based on the flow through apparatus reported in [59] cannot capture the dynamics of the complex mesalazine absorption behaviour shown in Fig. 7. Besides, the IVIVC developed in [59] rely on the percent absorbed *versus* time plots derived from the deconvolution method used, which calculates the *in vivo* absorption rate without a time constraint based on the F.A.T. concept and the multiple zero-order absorption stages of the PBFTPK models. In view of the present findings the validity of the IVIVC developed in [59] is questionable.

**Fig. 8:**
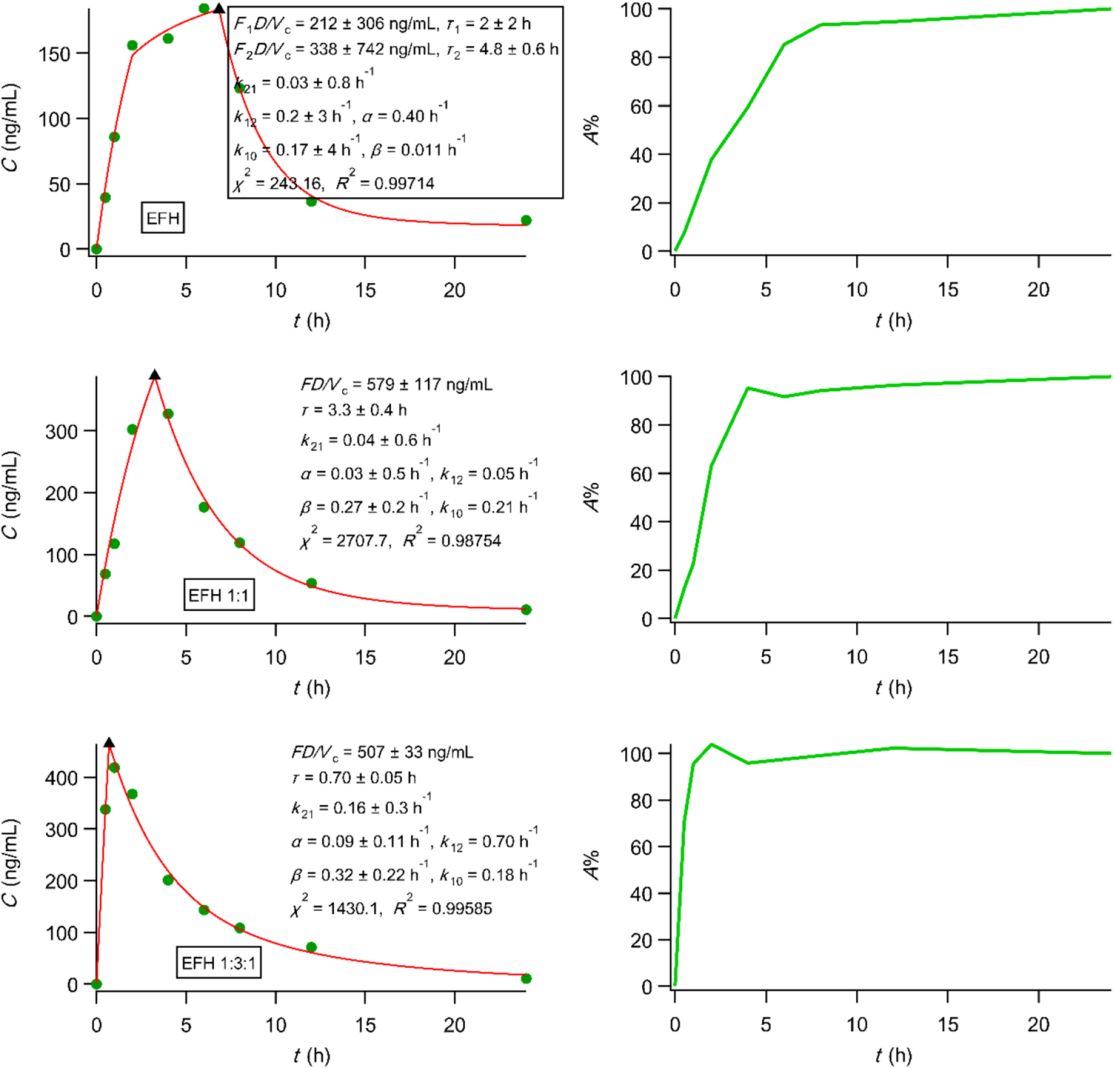
Best fitting results of PBFTPK models to efodipine data [60]; each plot is accompanied by the corresponding percent absorbed *versus* time curve calculated using Eq. 6 from Ref. [27]. The meaning of symbols can be found in [60].

We also analyzed the three sets of the *in vivo* data of efodipine hydrochloride [60] using PBFTPK models. Figure 8 shows the best fitting results for the three formulations examined using a one-compartment disposition model as well as the corresponding percent absorbed *versus* time plots. Only the crude drug exhibited two absorption stages with a total absorption duration 6.8 h indicating that efodipine (EFH) is absorbed in the small intestine with a rate of 106 ng mL^−1^ h^−1^ for 2 h, while the second absorption stage with a rate of 70 ng mL^−1^ h^−1^ lasts 4.5 h implying absorption from the colon too. On the contrary, EFH is absorbed from both dispersion formulations 1:1 and 1:3:1 in the small intestine following constant rates 175 ng mL^−1^ h^−1^ for 42 min and 704 ng mL^−1^ h^−1^ for 3.3 h, respectively. The dissolution EFH profiles of both dispersion formulations 1:1 and 1:3:1 reported in [60] are nonlinear even though a flow through dissolution device was utilized. Accordingly, IVIVC cannot be explored as suggested in the “IVIVC revised” article [28].

All in all, the PBFTPK modelling work together with the modified in terms of the F.A.T. percent absorbed *versus* time plots, open a new physiologically based avenue of research for IVIVC. It is hoped that the adoption of the novel approach and the new strategy in the development of generics will gradually change the pessimistic views prevailing in the IVIVC field of research [53, 61, 62].

#### Bioequivalence assessment

Typically, therapeutic equivalence is declared when two products are bioequivalent. All bioequivalence studies rely on the 90% confidence interval test of the logarithmically (ln) transformed ${\left[AUC\right]}_{0}^{\infty }$ and *C*_max_ mean values for the reference and test formulations; when the calculated 90% confidence interval for the ratio of (ln) transformed ${\left[AUC\right]}_{0}^{\infty }$ and *C*_max_ mean values lies between 80 and 125%, bioequivalence is declared. Although, the statistical tests have been changed through the years from the inception of bioavailability in 1977 in FDA, the metrics ${\left[AUC\right]}_{0}^{\infty }$ for the extent of absorption and *C*_max_ for the rate of absorption are used routinely since then [5]. The use of both parameters ${\left[AUC\right]}_{0}^{\infty }$ and *C*_max_ originates from the prevailing hypothesis of first-order absorption and the subsequent infinite absorption time [15]. However, many publications have invalidated the use of *C*_max_ as a rate metric in bioequivalence studies, e.g., [63–67]. We discuss below model-depended and model-independent (non-compartmental) approaches both based on the F.A.T. concept for the assessment of bioequivalence.

**Fig. 9:**
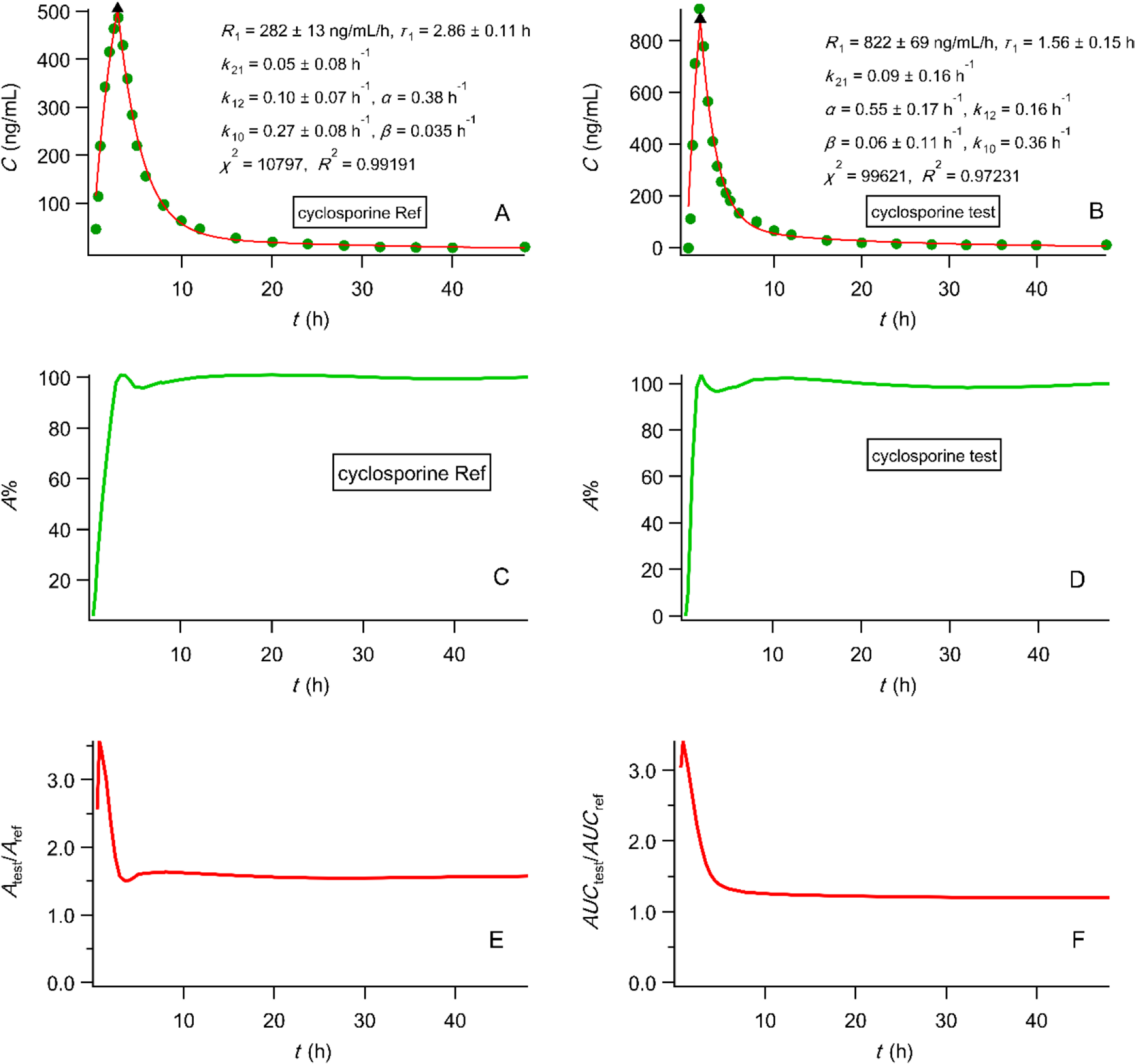
Model-depended approach: PBFTPK model fittings to cyclosporine data under fasted conditions [15] for reference (**A**) and test (**B**); R_1_ denotes the rate of input. Model independent approach: Percent absorbed *versus* time curves (calculated using Eq. 6 from Ref. [27]) for reference (**C**) and test (**D**) studied under fasted conditions [15] and the corresponding plot (**Ε**) for the ratio test/reference of the amount absorbed for the assessment of extent of absorption. The plot of the ratio (test/reference) of areas under the curve was calculated directly from the experimental data as a function of time (**F**).

i) Model-depended approach. When bioavailability was established by FDA in 1977, modelling in clinical pharmacology was in a pre-infancy period. Later, the development of non-linear mixed effect modelling approaches resulted in several relevant publications, e.g., [68–70]; the most recent publications in this field of research are focusing on what they call model-integrated evidence approaches for the analysis of bioequivalence data that have sparse sampling, e.g., [71]. These studies [68–71] as well as all other model-depended approaches *invariably and consistently utilize *${\left[AUC\right]}_{0}^{\infty }$* and C*_max_ *as pharmacokinetic end points* in accord with the regulatory guidelines. In the light of the analytical power and the physiological character of PBFTPK models and in line with the spirit of the MIDD initiative [6–8], we argue that a model depended approach based on the fitting results of the PBFTPK models to the experimental data can be used for the assessment of bioequivalence studies. We present an example of such an analysis in Fig. 9A and B. Table 2 shows the extent and rate of absorption metrics derived from the fitting of PBFTPK models to cyclosporine bioequivalence data under fasted conditions [15]. Τhe areas under the curves were calculated with numerical integration up to the last experimental point.

**Table 2:** Extent and Rate of Absorption Metrics and Their Estimates Derived from the Fitting Results of PBFTPK Models to Bioequivalence Data of Cyclosporine Studied Under Fasted Conditions [15]

	Metrics:	Extent of absorption		Rate of absorption		
Data set	Dose (mg)	${\left[AUC\right]}_{0}^{\infty }$ (h ng mL^−1^)	${\left[AUC\right]}_{0}^{\tau }$ (h ng mL^−1^)	*C*_max_ = *C*(*τ*) (ng mL^−1^)	Input rate (ng mL^−1^ h^−1^)	Duration (*τ*) (h)
Cyclosporine Ref fasted	300	2815	845	486	282 ± 1^a^ 324 ± 15^b^	2.86 ± 0.11^a^ 2.73 ± 0.08 ^b^
Cyclosporine Test fasted	180	3394	634	923	822 ± 69^a^ 1140 ± 165^b^	1.56 ± 0.15^a^ 1.37 ± 0.13^b^

^a^Derived from the fitting of PBFTPK models to the experimental data, Fig. 7A and B (model dependent approach)

^b^Derived from the analysis of the percent absorbed *versus* time plots, Fig. 7C and D (model independent approach)

Τhe higher bioavailability of the test formulation is obvious if one takes into account the different doses used. Correcting for dose, the ratios for the areas under the curve are $\frac{300}{180}\frac{{{\left[AUC\right]}_{0}^{\infty } }_{test}}{{{\left[AUC\right]}_{0}^{\infty }] }_{ref}}=2.0$, $\frac{300}{180}\frac{{{\left[AUC\right]}_{0}^{\tau } }_{test}}{{{\left[AUC\right]}_{0}^{\tau } }_{ref}}=1.25$. The estimate of the relative bioavailability of the two formulations is higher using the ${\left[AUC\right]}_{0}^{\infty }$ than the ${\left[AUC\right]}_{0}^{\tau }$, while in previous studies of theophylline [14] and digoxin [72] the two metrics ${\left[AUC\right]}_{0}^{\infty }$ and ${\left[AUC\right]}_{0}^{\tau }$ provided equal bioavailability estimates in accord with the F.A.T. concept since the absorption process(es) terminates at time *τ*. This difference should be attributed to the interindividual variations in the cyclosporine clearance and the high first-pass effect (gut metabolism) [73] of cyclosporine which is also affected by the significantly different input rates of the two formulations shown in Table 2. In contrast, both theophylline and digoxin do not exhibit first-pass effect, while theophylline is a class I drug and digoxin is used in micrograms and their absorption is completed in less than 1.5 h [14, 26]; therefore, the ${\left[AUC\right]}_{0}^{\tau }$ values correspond to the fraction of drug absorbed up to time *τ*, which is also the bioavailable fraction. The use of *C*_max_ as a rate parameter has been severely criticised [63–67] prior to the F.A.T. era. According to our findings [14], *C*_max_ represents the concentration of cyclosporine at the end of the absorption process, *C*(*τ*). In fact, the test/reference ratio for *C*_max_ is 486/923 = 0.52; this value is almost twice as high as the corresponding ratios of the input rates calculated from the experimental data with two methodologies based on the F.A.T. concept listed in Table 2, i.e., 282/822 = 0.34 and 324/1140 = 0.28. This is an additional [63–67] piece of evidence that *C*_max_ does not represent the rate of cyclosporine absorption. It should be emphasized that the input rates reported in Table 1 have rate units in full agreement with the spirit of rate as defined in the original FDA bioavailability document in 1977 [5]. Finally, the duration of cyclosporine absorption from the reference formulation is longer (2.86 ± 0.11 h) than from the test formulation (1.56 ± 0.15 h).

ii) Model-independent approach. The method relies on the modified Wagner-Nelson [24, 25] and Loo-Riegelman [26] techniques, which are used for the construction of percent absorbed *versus* time plot as described in [27]. The analysis of cyclosporine data, studied under fasted conditions in [15], is presented in Fig. 9C, D, E and F; in all cases, the areas under the curves were calculated with numerical integration. The slope of the percent absorbed *versus* time bilinear plots (Fig. 9C and D) for the two formulations, corresponds to the rate of input and has been proposed as the metric for the assessment of rate of absorption in [74]. The estimates of the input rate for the two formulations are listed in Table 2; quite similar results are obtained using the model-dependent and model independent methodologies since the pairwise statistical comparison of the input rates is not statistically significant. The constant ratio test/reference of the percent absorbed *versus* time plot at the plateau beyond the completion of cyclosporine absorption from the two formulations, Fig. 9E, corresponds to the relative bioavailability of the two formulations. An estimate for this constant ratio equal to 1.56 was calculated from the intersection of the back extrapolated horizontal line and the *y*-axis of Fig. 9E. This value can be corrected in terms of the doses used, 1.56 × (300/180) = 2.6. This estimate differs from the estimate 2.0 derived from the model-dependent approach. This difference should be attributed to the different methodology used in the two approaches for the numerical integration (trapezoidal rule) of the areas under the curves. In the model-dependent approach the areas were calculated directly from the experimental data up to the last time point and therefore an unbiased estimate was derived for the relative bioavailability, 2.0. In the model independent approach, the calculation relies first on the estimate of the elimination rate constant, *k*_10_ followed by an iterative calculation of drug concentration in the peripheral compartment, which affects the final estimate for the relative bioavailability. A visual check plot for the pharmacokinetic characteristics and the bioequivalence assessment can be based on Fig. 9 F, which shows the ratio of the areas of the two formulations calculated directly from the experimental data as a function of time; this plot shows clearly the end of the absorption processes and the slight decline as a function of time of the final limb of the curve indicating the larger clearance of the individuals receiving the test formulation throughout the elimination phase of the two formulations, i.e., beyond the completion of absorption process. The estimate of the ratio of the areas at the last datum point is 1.202, which if corrected in terms of dose is identical to the relative bioavailability estimate derived from the model-dependent approach, i.e., 1.202 × (300/180) = 2.0. However, the estimates for the duration of cyclosporine absorption from the reference formulation is longer (2.73 ± 0.08 h) than from the test formulation (1.37 ± 0.13 h). Overall, similar estimates for the duration of cyclosporine absorption are derived from the two methodologies, Table 2.

Important conclusions can be derived from this analysis concerning the assessment of extent and rate of absorption in the assessment of bioequivalence. Although the ${\left[AUC\right]}_{0}^{\tau }$ metric seems to be ideal for the assessment of the extent of absorption [14, 26], the present work underscores that ${\left[AUC\right]}_{0}^{\tau }$ should not be used when first-pass effect phenomena are operating and/or interindividual variations in the clearance of the two treatments are observed. The model dependent approach opens a new avenue for rate considerations in bioequivalence. This is so since the PBFTPK models provide the number, the magnitude and the duration for each one of the absorption stages. In parallel, the model independent approach provides an equally well estimation of the rate of absorption. In addition, the ratio of areas as a function of time plot gives an insight for the end of absorption process as well as the relative magnitude of clearance of the two treatments. When rate was introduced in 1977, the term was obscure, not well defined [75] and remained as such since a single fictitious first-order absorption rate constant was (is) used to quantify oral drug absorption. In the light of F.A.T. advances the concept of rate in bioequivalence studies should be reconsidered and the use of *C*_max_ as a rate metric should be discontinued.

## Regulatory Implications

All guidelines of regulatory Agencies rely on the dogma “Guidelines are scientifically based’. This work and the previous studies [12–15, 27, 38, 58, 68] on the F.A.T. concept, point to the fact that many scientific views require re-consideration and the use of PBFTPK models in pharmacokinetics and pharmacometrics instead of the first-order absorption models should be adopted. Plausibly, the PBFTPK models should be quoted among the modeling and simulation approaches in the “ICH M15 Guideline on general principles for model informed drug development”. This will gradually place an end to the perpetuation of infinite oral drug absorption fallacy in pharmacokinetics and pharmacometrics research as well as the relevant dossier submitted for approval to FDA and EMA. In the same vein, the Drug Agencies should consider the results of the present study for the guidelines associated with IVIVC, bioavailability and bioequivalence. Although these guidelines have been evolving for 50 years now and they are very well-established today, a dialogue should start in view of the physiologically relevant F.A.T. concept and the associated applications of the PBFTPK models delineated above.

## Conclusions

The current thinking in oral drug absorption is not in line with the finite time character of the drug processes taking place under *in vivo* conditions. The application of the physiologically sound F.A.T. concept coupled with the analysis of pharmacokinetic data with the relevant PBFTPK models are more akin to *in vivo* conditions and therefore capture the dynamics of drug absorption processes. These approaches can be applied successfully in all phases of drug/generics development and justify the changes required in the strategy of drug and generics development. Relevant regulatory changes are anticipated.

## Appendix

Equations 1A and 2A describe the drug concentration as a function of time following zero-order input kinetics for time *τ* assuming one-compartment model disposition

1A $$\begin{array}{cc}C\left(t\right)=\frac{FD}{{V}_{\text{d}}{k}_{\text{el}}\tau }\left(1-{\text{e}}^{{-k}_{\text{el}}t}\right)& \text{for }t \le \tau \end{array}$$

2A $$\begin{array}{cc}C\left(t\right)=C\left(\tau \right){\text{e}}^{-{k}_{\text{el}}\left(t-\tau \right)}& \text{for }t > \tau \end{array}$$

where *D* is the drug dose, *F* is the bioavailable fraction, *V*_d_ is the volume of distribution and *k*_el_ is the elimination rate constant. The amount of drug eliminated up to time *τ*, *Q*_el_(*τ*) is

3A $${Q}_{\text{el}}(\tau )={V}_{\text{d}}{\int }_{0}^{\tau }{k}_{\text{el}}C(t)\text{d}t={V}_{\text{d}}{\int }_{0}^{\tau }{k}_{\text{el}}\frac{FD}{{{V}_{\text{d}}k}_{\text{el}}\tau }\left(1-{\text{e}}^{-{k}_{el}t}\right)\text{d}t=\frac{FD}{\tau }\left(\tau -0\right)+\frac{FD}{{k}_{\text{el}}\tau }\left(1-{\text{e}}^{-{k}_{\text{el}}\tau }\right)=FD\left[1-\frac{1-{\text{e}}^{-{k}_{\text{el}}\tau }}{{k}_{\text{el}}\tau }\right]$$

Therefore, the amount of drug in the body at time *τ* is equal to $FD-{Q}_{\text{el}}(\tau )$. The corresponding area ${\left[\text{AUC}\right]}_{\uptau }^{\infty }$ is equal to

4A $${\left[\text{AUC}\right]}_{\tau }^{\infty }=\frac{FD-{Q}_{\text{el}}(\tau )}{CL}$$

which is Eq. 1 of the text.
